# Blocking of the High-Affinity Interaction-Synapse Between SARS-CoV-2 Spike and Human ACE2 Proteins Likely Requires Multiple High-Affinity Antibodies: An Immune Perspective

**DOI:** 10.3389/fimmu.2020.570018

**Published:** 2020-09-17

**Authors:** Indu Khatri, Frank J. T. Staal, Jacques J. M. van Dongen

**Affiliations:** ^1^Department of Immunology, Leiden University Medical Center, Leiden, Netherlands; ^2^Leiden Computational Biology Center, Leiden University Medical Center, Leiden, Netherlands

**Keywords:** ACE2, SARS-CoV, interaction-synapse, antibody, binding-affinity, felines, interface, IgA dimer

## Abstract

The pandemic of Coronavirus Disease 2019 (COVID-19) caused by SARS-CoV-2 has induced global eagerness to develop vaccines and therapeutics for treating COVID-19, including neutralizing antibodies. To develop effective therapeutic antibodies against SARS-CoV-2, it is critical to understand the interaction between viral and host's proteins. The human ACE2 (_h_ACE2) protein is the crucial target for the SARS-CoV's Spike protein that allows the virus to adhere to host epithelial cells. X-ray crystal structures and biophysical properties of protein-protein interactions reveal a large interaction surface with high binding-affinity between SARS-CoV-2 and _h_ACE2 (18 interactions), at least 15-fold stronger than between SARS-CoV-1 and _h_ACE2 (eight interactions). This suggests that antibodies against CoV-1 infection might not be very efficient against CoV-2. Furthermore, interspecies comparisons indicate that ACE2 proteins of man and cat are far closer than dog, ferret, mouse, and rat with significant differences in binding-affinity between Spike and ACE2 proteins. This strengthens the notion of productive SARS-CoV-2 transmission between felines and humans and that classical animal models are not optimally suited for evaluating therapeutic antibodies. The large interaction surface with strong affinity between SARS-CoV-2 and _h_ACE2 (dG−12.4) poses a huge challenge to develop reliable antibody therapy that truly blocks SARS-CoV-2 adherence and infection. We gauge that single antibodies against single epitopes might not sufficiently interfere with the strong interaction-synapse between Spike and _h_ACE2 proteins. Instead, appropriate combinations of high-affinity neutralizing antibodies against different epitopes might be needed, preferably of IgA-class for optimal and prolonged activity at epithelial layers of respiratory and intestine tracts.

## Introduction

As of June 2020, the coronavirus disease 2019 (COVID-19) pandemic has infected more than six million individuals with severe acute respiratory syndrome coronavirus 2 (SARS-CoV-2). SARS-CoV-2 belongs to the same family of *Coronaviridae* as SARS-CoV-1, which emerged in 2003. As both viruses belong to the same family and use similar modes of transmission to infect the host, research from the past 15 years on SARS-CoV-1 could be extended to SARS-CoV-2 (1, 2). For example, the interaction between the angiotensin-converting enzyme 2 (ACE2) protein in humans (_h_ACE2) and the Spike (S) protein in SARS-CoV's (_CoV′s_S) is known from the 2003 outbreak (3, 4). This is in contrast to the related Middle East respiratory syndrome (MERS)-CoV virus, known from the 2012 outbreak (5), which has a S protein, that does not bind to _h_ACE2, but to dipeptidyl peptidase-4 (DPP4), also known as the CD26 receptor (6, 7). The discovery of the S-protein binding of the SARS-CoV-1 virus to the _h_ACE2 protein was important to understand the mode of interaction of the SARS-CoV-2 virus with the host cells. Later, the interaction between the receptor binding domain (RBD) of the S1 domain in the viral S protein and the ACE2 receptor binding domain was X-ray crystallized (8), similar to the interaction between _CoV1_S and _h_ACE2. Several bound and unbound conformations of this protein-protein interaction have been crystallized to understand the affinity of the interactions between _CoV1_S and _h_ACE2 proteins (8–13). These structures have enabled researchers to assess the impact of mutations on the structural and functional properties of the interactions between _CoV′s_S and _h_ACE2 proteins in different species (3, 14). The sequence information of the ACE2 gene, supported by the structural properties has also been used to understand the possibility of transmission of the virus between different species, especially vertebrates (14).

Previously, the similarity of ACE2 receptors between human and non-human primates has been reported (14). However, the social and economic position of these animals have not been considered. For example, several reports have lately emerged with transmission of COVID-19 infection from humans to their pets and domesticated animals (14–17). These reports have led to questions about SARS-CoV-2 transmission in a vice-versa situation (i.e., from animals to humans). With the transmission of infection from minks to a human at mink farm in Netherlands (https://www.government.nl/latest/news/2020/05/19/new-results-from-research-into-covid-19-on-mink-farms) and COVID-19 infections at eight mink farms (https://www.government.nl/latest/news/2020/06/01/initial-investigation-results-infections-at-three-more-mink-farms) makes this question even stronger and furthermore strengthens our argument that economical and societal organization plays an important role. Furthermore, although the ACE2 receptor of cats is not completely identical to humans, of all domesticated/pet animals, the cat's ACE2 receptor is closest to human and indeed infected cats show symptoms similar to humans (www.rivm.nl/en/novel-coronavirus-covid-19/pets). Consequently, cats pose a more serious but less-mentioned threat for animal to human transmission in many societies, especially those cats that wander around and are in close contact with many different families. However, this is not as straight-forward as it seems, because viral dosage plays an important role in such transmission.

Importantly, several options are proposed to treat or mitigate COVID-19 disease (18–20). Increasingly, convalescent serum of individuals recovered from COVID-19 is becoming a treatment of choice (21, 22). Understanding the exact nature of the _CoV′s_S protein interaction with the _h_ACE2 receptor molecule is essential to understand how the treatment with passive-immunization may be effective in humans in neutralizing the virus from adhering and entering the epithelial target cells, thereby controlling the infection. For example, neutralizing antibodies (nAbs) developed against CoV-1, have been tested against SARS-CoV-2, but the neutralization capability of these nAbs against SARS-CoV-2 is lower than for SARS-CoV-1 (23, 24). Recently, a human monoclonal antibody was proposed to be effective against COVID-19 that targets a general receptor binding domain on SARS viruses (25). However, the exact epitope of that antibody on the viral domains has not been mapped.

Here, we wish to draw the attention of immunologists to the interaction interface between _CoV2_S and _h_ACE2 proteins, which is substantially larger than in _CoV1_S-_h_ACE2 interactions. As immunotherapy with blocking antibodies against the S viral protein can significantly contribute to combating COVID-19 infection, it will be critical to identify the appropriate epitopes on the viral S protein, to be blocked with a combination of two or more different high-affinity antibodies.

## _CoV2_S-_h_ACE2 Interaction Synapse Is Larger and Stronger Than _CoV1_S-_h_ACE2

**Figure 1:**
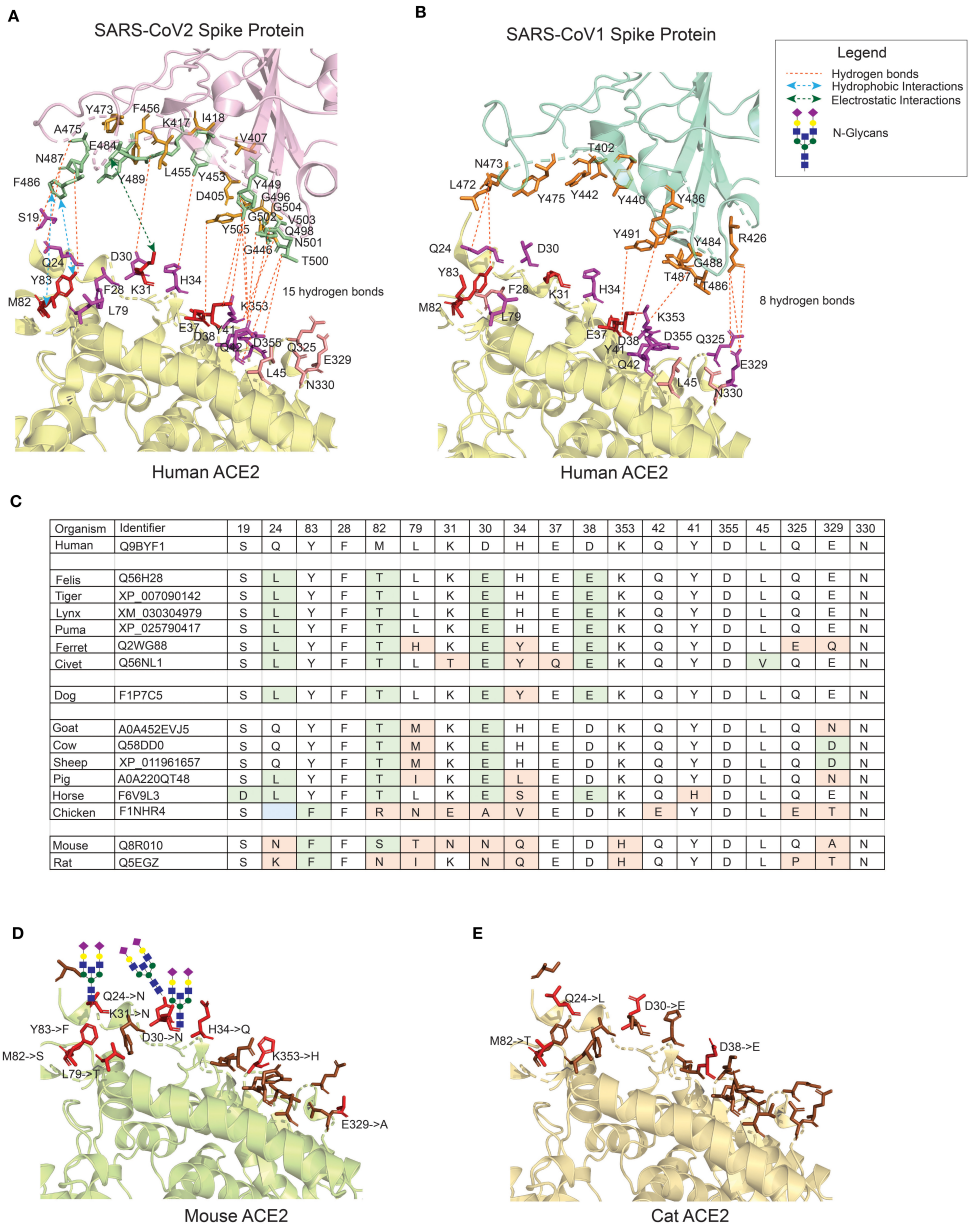
The interface of ACE2 protein in different organisms with important amino-acid residues for interacting with the Spike (S) protein of SARS-CoV viruses. **(A)** The interacting interface between _h_ACE2 protein and _CoV2_S (PDB ID: 6LZG). The residues in _h_ACE2 proteins are colored in red, magenta and pink. Red residues are the most important and pink the least. The interacting residues in _CoV2_S are colored in green and orange colors where green residues are the important interacting residues. The distance between _CoV2_Spike and _h_ACE2 proteins is increased to better visualize the residues and the interactions. **(B)** The interacting interface between _h_ACE2 protein and _CoV1_S (PDB ID: 2AJF). Red residues on _h_ACE2 protein are important residues for maintaining the interaction between _h_ACE2 and _CoV1_S proteins. All the residues in _CoV1_S interacting with _h_ACE2 are colored in orange. The distance between _CoV1_Spike and _h_ACE2 proteins is increased to better visualize the residues and the interactions. Hydrogen bonds **(A,B)** as described in the structures of these interaction (PDB ID: 6LZG and 2AJF) and electrostatic and hydrophobic bonds in _CoV1_S-_h_ACE2 interaction are depicted from Brielle et al. (4). **(C)** The positions mutated in ACE2 proteins in selected vertebrates that are either pets, domesticated or live in vicinity of humans. The mutated residues are shaded with green or orange background. The green background represents mutations resulting in similar property residue i.e. polar -> polar or non-polar -> non-polar. The orange background represents mutations resulting in changes in the residue property i.e. polar -> non-polar. **(D)** The interface of mouse's ACE2 protein. Red color residues represent the mutated residues. Three mutations on the left interacting region has resulted in an Arginine, introducing a glycan site. The basic structure of glycan is shown on the sites. **(E)** The interface of cat's ACE2 protein. The red color residues represent the mutated residues. The mouse's and cat's ACE2 structure are modelled with modeller-9.24 (30) using 6LZG as a template.

_h_ACE2 receptors are widely expressed in multiple tissues i.e., nasopharynx, trachea, bronchi, and lung, but also in gut, kidney, vessels, heart, testis and brain (26–28). These receptors are not only the door to virus-entry but also conductor of pathophysiological reactions associated with the clinical features of the disease (29). The entry of SARS viruses is mediated by efficient binding of the viral S protein to the _h_ACE2 receptor. Though the receptor binding domain (RBD) of S proteins in SARS-CoV-1 and SARS-CoV-2 share high homology at both sequence (88%) and structure level, the interaction surface of _CoV2_S by comparison is larger than _CoV1_S (Figures 1A,B). The residues in the _h_ACE2 protein that were found to be crucial in maintaining the interaction with the RBD domain by Li et al. (31) are shown in red for _CoV2_S (Figure 1A) and _CoV1_S (Figure 1B). These residues mostly share polar bonds with multiple tyrosine residues present at the interacting interface of the viral S protein. The residues marked in magenta (Figures 1A,B) are equally important in maintaining multiple weaker interactions (hydrogen bonds), which altogether results in strong protein-protein interactions (PPI) (32, 33). Consequently, 18 interacting PPI pairs are found between _CoV2_S and _h_ACE2 within 1773Å^2^ as compared to only eight in the _CoV1_S-_h_ACE2 interaction (Figures 1A,B). As a consequence, the binding affinity of _CoV2_S-_h_ACE2 is 15-fold higher than that of _CoV1_S-_h_ACE2 [Kd of 8.30E-10 M as compared to Kd of 1.20E-08 M in _CoV1_S-_h_ACE2; as calculated by PRODIGY (34)].

The remarkably tyrosine-rich interacting surface of the viral S protein implies a role for evolutionary optimizing selection mechanisms to stepwise enhance the affinity and specificity of the _CoV2_S protein, thereby increasing the molecular interaction with _h_ACE2 (35). However, the sudden start of the SARS-CoV-2 pandemic in Q4 of 2019 can hardly have permitted evolutionary optimizing selection processes with stepwise accumulation of tyrosine residues at positions appropriate for increased _CoV2_S-_h_ACE2 affinity.

Within the synapse between the _CoV2_S protein and the _h_ACE2 receptor, one can discern two main interaction regions: one around amino-acid positions 24–30 and another around 42–53 in _h_ACE2 receptor corresponding to 471–486 and 496–505 amino-acid positions in _CoV2_S protein with additional residues in 404–416 and 446–456 amino-acid positions contributing to the adhesion synapse (Figure 1A). Based on these structural considerations, we conclude that the high-affinity interaction between the S protein and ACE2 receptor is caused by multiple interacting residues in _CoV2_S-_h_ACE2 PPI, which form the strong (multi-epitope) adhesion synapse between the viral surface and host's epithelial layer.

## Classical Animal Models Are Not Optimally Suited for Evaluating Therapeutic Antibodies In SARS-CoV's Infection Models

The receptor binding domain of viral S proteins may mutate to modify binding affinity for ACE2, and hence lead to differential infection potentials in humans or other hosts. For example palm civet was believed to be the intermediate host between bats and humans for SARS-CoV-1 (5). The intermediate host for SARS-CoV-2 is not known yet, although pangolins have been proposed as a possible reservoir (36). The sequence and structural features of the ACE2 proteins of different species can predict their ability to bind to the viral S protein and hence can provide insight into the potential virus transmission. ACE2 protein sequences from different vertebrate species have been assessed to predict their ability to bind _CoV2_S (14, 37). However, these studies did not take into account the societal position of these vertebrates. For example, owing to similar ACE2 proteins, non-human primates are found to have similar binding affinity to the virus as that of humans. Due to the almost non-existent physical and societal interaction between humans and non-human primates, the chance of transmission between these two groups is limited. The viral load plays an important role in transmitting the COVID-19 infection and henceforth the severity of infection symptoms (38, 39). The transmission rate will be higher between humans and animals that are truly domesticated by humans, especially pets that live closely together (in the same house) with their owners and other individuals, who (un)wantedly are in close contact. Several reports of transmission of infection from humans to cats and tigers and from cats to cats have been reported recently (16, 40–44). And a vice-verse transmission i.e., from cats to humans is also possible owing to cat's societal and economical value especially in the western society.

To better understand animal-human transmission, we have compared the variant residues in the ACE2 proteins in multiple organisms which are either close to humans or are domesticated (Figure 1C). The variant amino acid residues in the ACE2 proteins of these organisms are predicted to have impact on the interaction, if the variant results in changed properties of the residue (residues in orange background; variants in green do not result in changed properties). The 3D structure of the ACE protein is generated using comparative modeling for mouse (Figure 1D) and cat (Figure 1E) with a template of X-ray crystallographic structure 6LZG (8). The number of variant residues in mouse is higher than in cats and the introduction of Arginine in the binding domain results in introduction of N-glycosylation sites at positions Q24, D30, and K31 (Figure 1D). The overall binding site of the murine ACE2 protein is quite different from human, hampering the interaction with the viral S protein, although not completely. This immediately points to the insufficiency of mouse models for assessing molecular and therapeutic intervention studies for SARS-CoV's infection model. The high similarity of the _h_ACE2 protein with ACE2 receptors in felines make them a better model to assess coronavirus infection models. Whereas, ferrets and civets, among felines, are currently used as COVID-19 infection models, cat's ACE2 is much more similar to _h_ACE2 (see Figure 1C for details). Furthermore, cats have been shown to be permissive of the virus (16), the large nasopharynx surface area in cats can lead to variations in the time of entry and permissiveness of virus as compared to humans.

In conclusion, the similar ACE2 receptors of cats and man, with several cases of transmission between cats and humans documented (16, 40–44), and their close social relationship may pose a threat of further transmission. Moreover, the extended nasopharyngeal anatomy in cats with a suboptimal immune response can potentially make them a reservoir of the virus, which can pose a threat of transmission to humans. This might be an inconvenient message in many western societies. Most importantly, the many differences in ACE2 molecules between man and laboratory animals, make classical animal models not optimally suited for evaluating therapeutic antibodies in SARS-CoV-2 infection models.

## Neutralizing Antibodies Against SARS-CoV-2

Convalescent plasma of COVID-19 survivors is being used as a direct approach to combat SARS-CoV-2 infection in severe COVID patients (21, 22). This is one of the simplest approaches for using polyclonal neutralizing antibodies from convalescent patients, providing passive immunization. Other antibody therapies considered for COVID-19 include polyclonal normal IgG, hyperimmune IgG and monoclonal antibodies from COVID-19 survivors (45–47). The neutralizing antibodies are a subset of antibodies present in convalescent plasma that reduces viral infectivity by binding to the epitopes of viral particles and hence block the adherence of virus to host cells. The therapy is effective yet unpredictable due to the genetic and immune variability between different individuals. As adherence of SARS-CoV virus to host cells is mediated by interaction of the RBD domain of the viral S protein with host's ACE2 receptor, the viral S protein becomes a major target for developing the neutralizing antibodies. The neutralizing monoclonal antibodies targeting SARS-CoV epitopes are reviewed by Shanmugaraj et al. (48). Most of these neutralizing monoclonal antibodies indeed recognize the receptor binding region in the S1 domain of viral S protein for SARS-CoV-1 (48). Two neutralizing antibodies CR3022 and CR3014 bind to the S2 domain of the S viral protein of SARS-CoV-1 and recognize different epitopes (49). The cocktail of these two antibodies appeared more powerful in neutralizing CoV-1 virus as compared to CR3022 only. Therefore, the data from SARS-CoV-1, already indicate that a single antibody might not be sufficient to combat the SARS-CoV-2 infection efficiently. Although the CR3022 and CR3014 antibodies were able to 100% neutralize SARS-CoV-1 infection, the same cocktail will not be equally effective against SARS-CoV-2 infection (24). This argument is supported by the larger interface of the _CoV2_S-_h_ACE2 protein-protein interaction (Figure 1) and also >15-fold higher binding affinity of the _CoV2_S-_h_ACE2 interaction [Kd of 8.30E-10 M as compared to 1.20E-08 M in _CoV1_S-_h_ACE2; as calculated by PRODIGY (34)]. The surface plasma resonance experiments by Wang et al. showed 4-fold higher affinity of _CoV2_S-_h_ACE2 as compared to _CoV1_S-_h_ACE2 (8), while 10 to 20-fold higher affinity of _CoV2_S-_h_ACE2 as compared to _CoV1_S-_h_ACE2 has been mentioned previously (23, 50). Of note, the affinity for binding of _CoV2_S to _h_ACE2 proteins is higher than many other protein-protein interactions in nature, including one of the highest antibody affinities (namely anti-TSH-R antibodies, which is 7.2E-09 M Kd) and far higher than T-cell receptor interactions with Major Histocompatibility Complex molecules (2.00E-06 M Kd).

## Currently Reported Antibodies Need a Partner Antibody to Completely Hinder _CoV2_S-_h_ACE2 Interaction

**Figure 2:**
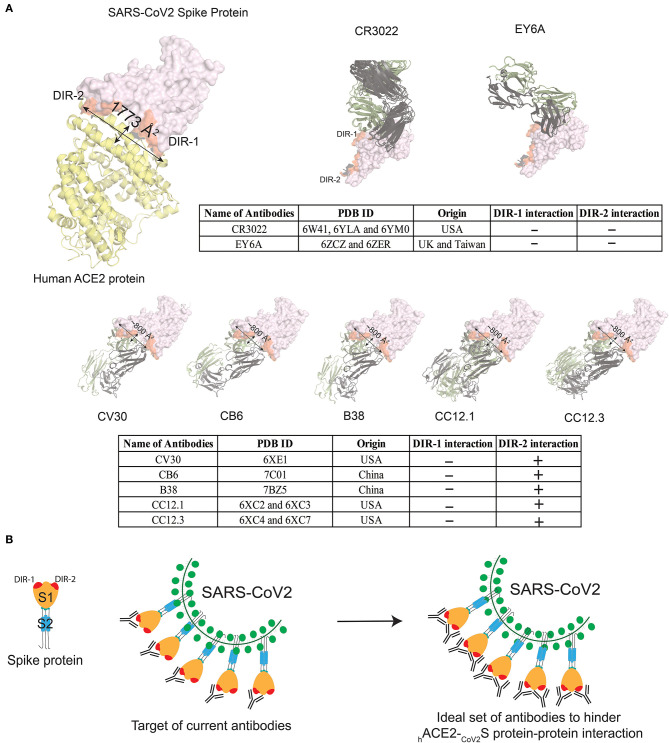
Currently known antibodies targeted against _CoV2_S protein do not hinder the complete interaction site between _CoV2_S and _h_ACE2 proteins. **(A)** Visualizing interacting surface between _CoV2_S and _h_ACE2 proteins (PDB ID: 6LZG). Two highly dominant interacting regions (DIR) in _CoV2_S protein are referred as DIR-1 and DIR-2. Two antibodies in the top panel bind to Spike protein epitopes that do not interact with _h_ACE2. Multiple X-ray crystallographic structures for one antibody were aligned in PyMOL (59). Five antibodies in the bottom panel from four different studies interact with the interaction region between _CoV2_S and _h_ACE2 proteins. These five antibodies interact with the DIR-2 region, but not with residues in the DIR-1 region. The surface area of the interacting region in _CoV2_S protein is 1,773Å^2^ surface area, whereas an antibody can cover 600–900 Å^2^ surface. **(B)** Schematic diagram to explain how the currently known antibodies can completely hinder _CoV2_S-_h_ACE2 interaction with the help of a partner antibody.

Recently, several human-origin monoclonal antibodies with neutralizing capacity were isolated from convalescent patients, which were further tested in mouse models for their therapeutic potential against SARS-CoV-2 (12, 51–57). For several of these antibodies, the 3D structures are available (12, 51, 52, 54, 55, 58). Moreover, the anti-SARS-CoV-1 high-affinity antibodies i.e., CR3022 and EY6A, which also recognize SARS-CoV-2, are not able to hinder the _CoV2_S-_h_ACE2 protein-protein interaction, because the recognized epitopes are located outside the adhesion synapse (Figure 2A). Five other anti-RBD-antibodies (RBD-CV30, RBD-CB6, RBD-B38, RBD-CC12.1, and RBD-CC12.3) have been more effective in interfering with the _CoV2_S-_h_ACE2 protein-protein interaction, albeit that they target similar epitopes (Figure 2A). These five high-affinity antibodies are not able to completely block the adhesion synapse between the _CoV2_S and _h_ACE2 proteins, because they bind to only one of the two dominant interaction regions (DIR-1 and DIR-2) in _CoV2_S-_h_ACE2 protein-protein interaction (Figure 2A). Consequently, the DIR-1 region of the _CoV2_S protein is still accessible for interaction and therefore a partner antibody might be required to completely block the adhesion synapse (Figure 2B). It is striking that all reported antibodies with epitope mapping target the DIR2 region, which may represent an immunodominant epitope. If this turns out to be the case, therapeutic mixtures of antibodies should be constructed to include antibodies that do not only target DIR-2, but DIR-1 as well. This notion might well be critically important for design of vaccines, which aim primarily at blocking the _CoV2_S-_h_ACE2 adhesion synapse to prevent infection.

## Several Factors Influence the Functional Aspects of Therapeutic Antibodies

Highly specific antibodies with high affinity are relevant for therapeutic application in COVID-19 patients. However, the functionality of such antibodies will be governed by several factors i.e., variant form of the viral S protein, ACE2 receptor, Fc glycosylation, Fc receptors and the antibody class (IgM, IgG1, IgG2, IgG3, IgG4, and/or IgA1 and IgA2). A recent report shows the role of D614G mutation variant of the Spike protein which is related to the infectivity of the SARS-CoV-2 (60), however, the precise meaning remains unclear (61). Moreover, the effect of the mutated epitopes in _CoV2_S protein on the effectiveness and affinity of the above-mentioned neutralizing antibodies will be interesting to understand. The ACE2 variants in different species are directly related to the level of infectivity, but no statistically significant mutation has been reported in the _h_ACE2 protein worldwide, so far (62). We believe that the ACE2 protein is not yet under selection pressure, as the COVID infections are recent encounters to the human populations. Of course, it will be very interesting to know how different populations will adapt genetically to these infections in the future, but evolutionary selection of the ACE2 receptor will be a very long-term adaptation to the COVID-19 infection.

The antibody itself can impact its therapeutic potential for example by post translational mechanisms i.e., Fc glycosylation can impact the pharmacokinetics and pharmacodynamics of the antibodies (63, 64). Also, the class-specific antibody response i.e., IgM, IgG, or IgA, impacts the functioning of the antibodies, because of the significant differences between the effector functions of antibodies of different classes. This does not only concern their complement binding and Fc receptor binding, but also other typical characteristics such as the efficient transport of IgA dimers through epithelial layers and their survival in mucosal lining fluid of the epithelial layers in respiratory tract and intestine. In most infection and vaccination studies, serum antibody analysis is focused on antigen-specific antibodies of IgM and IgG class, rarely IgA class. Importantly, we consider a major role of epithelium-linked IgA dimer antibodies because the virus enters *via* the mucosal route where the IgA antibody response is ideally a first line antibody defense mechanism of the immune system in blocking the virus to adhere to the epithelial layers. In line with these considerations, Sterlin et al. recently showed that the IgA response dominates the early neutralizing antibody response to COVID-19 infection and IgA response contributes more to virus neutralization as compared to the IgG response (65). These insights challenge the vaccination strategies where most of the vaccines are developed to raise IgG responses which may not provide optimal protection at the mucosal surfaces in respiratory infections, such as SARS-CoV-2 infections. The intranasal vaccination strategy and IgA-based therapeutic antibodies for the influenza infection (66, 67) and intranasal administration of a MERS-derived vaccine (68) clearly exploit the beneficial role of IgA antibodies at epithelial layers. Concludingly, IgA should be preferred over IgG-based responses in vaccination strategies and antibody treatment against COVID-19 infection.

## Concluding Remarks

Since the binding affinity of S viral proteins to mouse ACE2 receptors is less than half as compared to humans, non-human primates and cats (69), the antibodies raised in mouse models will not be equally effective in humans. Similar binding efficiency of cat's and human's ACE2 receptor with _CoV2_S viral protein makes the felines a better model to assess therapeutic and molecular research for COVID-19 infection. In this respect, caution should be given to cats as a frequently ignored reservoir of the virus.

To prevent and/or disrupt the unusually high-affinity of the _CoV2_S-_h_ACE2 interaction synapse, which contains at least two high-affinity interactions sites, one can predict that a single monoclonal “therapeutic” antibody is likely insufficient. Consequently, proper epitope mapping of therapeutic antibodies is needed, which should preferably target multiple tyrosine residues in the binding region that result in highly interactive polar bonds between viral S proteins and ACE2 receptors. Current antibodies are potent to cover the dominant interaction region (DIR)-2, whereas a partner antibody can completely hinder the _CoV2_S-_h_ACE2 interaction. Hence, passive immunization for COVID-19 infection should be composed of mixtures of different high-affinity antibodies that target different sites of the adhesion synapse between the _CoV2_S and _h_ACE2 proteins. Consequently, a set of high-affinity antibodies, preferably of IgA-class, recognizing multiple different epitopes of the synapse-region of the _CoV2_S protein are needed for an optimal and prolonged activity at epithelial layers of respiratory and intestine tracts. This notion should not only be considered in antibody-based therapies but also in the design of SARS-CoV-2 vaccine strategies.

## Data Availability Statement

All datasets presented in this study are included in the article.

## Author Contributions

IK performed the sequence and structure analysis in this perspective. All authors formulated the research question/premise, conducted research, literature reviews, wrote, and approved the manuscript.

## Conflict of Interest

JD is the founder of the EuroClonality and EuroFlow Consortia, one of the inventors on the EuroClonality-owned patents, and EuroFlow-owned patents, which are licensed to Invivoscribe, BD Biosciences or Cytognos; these companies pay royalties to the EuroClonality and EuroFlow Consortia, respectively, which royalties are exclusively used for sustainability of these consortia, and reports an Educational Services Agreement with BD Biosciences and a Scientific Advisory Agreement with Cytognos to LUMC. The remaining authors declare that the research was conducted in the absence of any commercial or financial relationships that could be construed as a potential conflict of interest.
