# Corrigendum: Blocking of the High-Affinity Interaction-Synapse Between SARS-CoV-2 Spike and Human ACE2 Proteins Likely Requires Multiple High-Affinity Antibodies: An Immune Perspective

**DOI:** 10.3389/fimmu.2021.659375

**Published:** 2021-04-14

**Authors:** Indu Khatri, Frank J. T. Staal, Jacques J. M. van Dongen

**Affiliations:** ^1^ Department of Immunology, Leiden University Medical Center, Leiden, Netherlands; ^2^ Leiden Computational Biology Center, Leiden University Medical Center, Leiden, Netherlands

**Keywords:** ACE2, SARS-CoV, interaction-synapse, antibody, binding-affinity, felines, interface

In the original article, there was a mistake in **Figure 1** as published. In the structure of the CoV2 Spike protein, at amino-acid position 501, G (Gly) was erroneously indicated and should be N (Asn). In the CoV1 Spike protein, Y (Tyr) position was erroneously mentioned at 477 which is position 475. The corrected **Figure 1** appears below.

**Figure 1:**
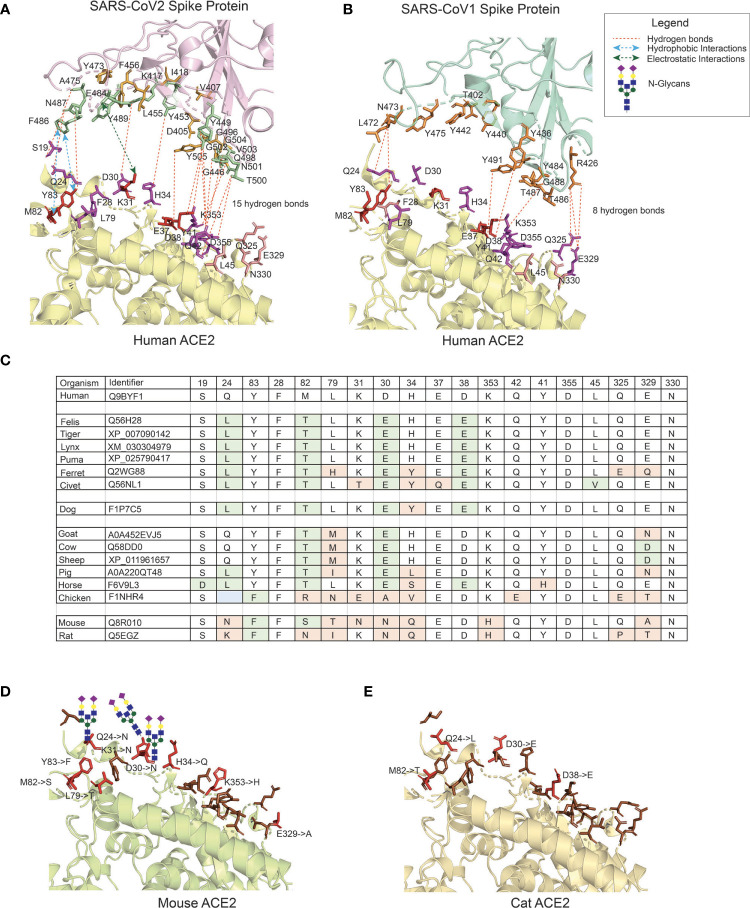
The interface of ACE2 protein in different organisms with important amino-acid residues for interacting with the Spike (S) protein of SARS-CoV viruses. **(A)** The interacting interface between _h_ACE2 protein and _CoV2_S (PDB ID: 6LZG). The residues in _h_ACE2 proteins are colored in red, magenta and pink. Red residues are the most important and pink the least. The interacting residues in _CoV2_S are colored in green and orange colors where green residues are the important interacting residues. The distance between _CoV2_Spike and _h_ACE2 proteins is increased to better visualize the residues and the interactions. **(B)** The interacting interface between _h_ACE2 protein and _CoV1_S (PDB ID: 2AJF). Red residues on _h_ACE2 protein are important residues for maintaining the interaction between _h_ACE2 and _CoV1_S proteins. All the residues in _CoV1_S interacting with _h_ACE2 are colored in orange. The distance between _CoV1_Spike and _h_ACE2 proteins is increased to better visualize the residues and the interactions. Hydrogen bonds (1A and 1B) as described in the structures of these interaction (PDB ID: 6LZG and 2AJF) and electrostatic and hydrophobic bonds in _CoV1_S-_h_ACE2 interaction are depicted from Brielle et al. (4). **(C)** The positions mutated in ACE2 proteins in selected vertebrates that are either pets, domesticated or live in vicinity of humans. The mutated residues are shaded with green or orange background. The green background represents mutations resulting in similar property residue i.e. polar -> polar or non-polar -> non-polar. The orange background represents mutations resulting in changes in the residue property i.e. polar -> non-polar. **(D)** The interface of mouse’s ACE2 protein. Red color residues represent the mutated residues. Three mutations on the left interacting region has resulted in an Arginine, introducing a glycan site. The basic structure of glycan is shown on the sites. **(E)** The interface of cat’s ACE2 protein. The red color residues represent the mutated residues. The mouse’s and cat’s ACE2 structure are modelled with modeller-9.24 (65) using 6LZG as a template.

The authors apologize for this error and state that this does not change the scientific conclusions of the article in any way. The original article has been updated.

